# Torque Teno Virus Control by the Classical Pathway of Complement Activation—A Retrospective Analysis From a First‐in‐Human Trial Utilizing Sutimlimab

**DOI:** 10.1002/jmv.70039

**Published:** 2024-11-06

**Authors:** Sebastian Kapps, Jakob Mühlbacher, Dorian Kulifaj, Sophie Courjal, Farsad Eskandary, Martin Schiemann, Bernd Jilma, Georg A. Böhmig, Gregor Bond, Markus Wahrmann

**Affiliations:** ^1^ Department of Medicine III, Division of Nephrology and Dialysis Medical University of Vienna Vienna Austria; ^2^ Department of Surgery, Division of Visceral Surgery Medical University of Vienna Vienna Austria; ^3^ bioMérieux SA, Parc Technologique Delta Sud Verniolle France; ^4^ Department of Clinical Pharmacology Medical University of Vienna Vienna Austria

**Keywords:** classical pathway, complement, immunosuppression, sutimlimab, Torque Teno virus

## Abstract

Torque Teno virus (TTV) load is linked with the functionality of its host's immune system and has been proposed as a potential monitoring tool for immune‐modulating therapy. However, the immunological mechanisms of TTV control are incompletely understood. To assess the effect of the classical complement pathway on TTV, 64 healthy volunteers and 10 kidney transplant recipients treated with the anti‐C1s antibody sutimlimab were analyzed for serum TTV copy numbers (c/mL) by qPCR. Overall, a correlation was observed between the decrease in complement activity caused by sutimlimab and the TTV load increase (*ρ* = −0.367, *p* < 0.001). Subgroup analysis indicated a trend toward TTV load increase in healthy volunteers following the highest sutimlimab dose compared to baseline (100 mg/kg body weight; median 3.5 log_10_ c/mL, interquartile range [IQR] 2.8–4.4 vs. 2.9 log_10_ c/mL, 0.8–3.5; *p* = 0.063). Administering multiple lower doses (30 mg/kg) also showed a trend toward TTV load increase in healthy volunteers (1.8 log_10_ c/mL, 0–2.3 vs. 1.9, 1.3–2.8; *p* = 0.054) and a significant increase in transplant recipients (3.5 log_10_ c/mL, 3.0–6.1 vs. 4.1, 3.5–6.4; *p* = 0.004). This report suggests a role for the classical complement pathway in controlling TTV load.

**Abbreviations**
AMR: antibody‐mediated rejection
C1, C1q, C1r, C1s, C3, C4: complement component 1, 1q, 1r, 1s, 3, 4
c/mL: copies per milliliter
CP: classical pathway
DSA: donor‐specific antibodies
HLA: human leukocyte antigen
IQR: interquartile range
MFI: mean fluorescence intensity
NK cells: natural killer cells
(q)PCR: (quantitative) polymerase chain reaction
TTV: Torque Teno virus

## 1 Introduction

Torque Teno viruses (TTV), classified under the genus *Alphatorquevirus* within the family *Anelloviridae*, comprise a group of closely related, nonpathogenic viruses prevalent in approximately 90% of healthy humans [1]. The PCR‐based quantification of TTV has been proposed to serve as a measure of the degree of immunosuppression in transplant recipients [2] and patients with autoimmune, infectious, and oncological diseases [3]. Immunocompetent hosts usually show chronic low‐level DNAemia, while immunosuppressed patients show a TTV load that is several log steps higher. Consequently, monitoring TTV load in blood has been demonstrated to help estimate the risk of opportunistic infections and allograft rejection in transplant recipients [4, 5], surveil the course of treatment in autoimmune [6] and oncological diseases [7], and monitoring respiratory TTV load may help estimate the risk of intensive care admission in patients with coronavirus disease 2019 [8].

It is well‐established that T cells and NK cells play a crucial role in the host defense against DNA viruses [9], a concept that has also been proposed for TTV. B cells have also been suggested to contribute specifically to TTV control [6]. However, data regarding the innate immune compartments controlling TTV load are scarce [10]; in particular, no studies have examined the role of the complement system thus far.

Assuming the presence of type G or M immunoglobulins targeting the open reading frame proteins of TTV [11], which activate the classical pathway of complement, it is imaginable that this activation could contribute to TTV control. An early step in classical pathway (CP) activation is the formation of the C1 complex, consisting of multiple subcomponents C1q, C1r, and C1s. Sutimlimab is a humanized monoclonal antibody binding and neutralizing the complement component C1s, thus abrogating the activation of downstream complement components like C4 and C3 [12]. The present study comprised a retrospective analysis of TTV load in biological specimens from subjects enrolled in a first‐in‐human trial designed to evaluate the safety and tolerability of sutimlimab, including both healthy subjects and kidney transplant recipients [13, 14, 15].

## 2 Materials and Methods

### 2.1 Trial Design

This retrospective study was performed in the context of a randomized, double‐blinded, placebo‐controlled, first‐in‐human trial designed to evaluate the safety and tolerability of sutimlimab. The phase 1 study is described in detail elsewhere [14, 15] and in the Supporting Information S6: Materials and methods. The main inclusion and exclusion criteria were age ≥ 18 years and any chronic illness or infection within the past 30 days, respectively. The trial consisted of three parts: in part A, 48 healthy adult volunteers were assigned to seven cohorts and received single ascending doses of sutimlimab or placebo (3:1 randomization in each cohort) as follows: cohort 1 (*n* = 4; 0.3 mg/kg), cohort 2 (*n* = 4; 1 mg/kg), cohort 3 (*n* = 8; 3 mg/kg), cohort 4 (*n* = 8; 10 mg/kg), cohort 5 (*n* = 8; 30 mg/kg), cohort 6 (*n* = 8; 60 mg/kg), and cohort 7 (*n* = 8; 100 mg/kg; Supporting Information S1). In part B, 16 volunteers received four weekly doses of sutimlimab or placebo (3:1 randomization in each cohort) as follows: cohort 8 (*n* = 8; 30 mg/kg) and cohort 9 (*n* = 8; 60 mg/kg; Supporting Information S1). In part C, 10 kidney transplant recipients with biopsy‐proven antibody‐mediated rejection (AMR) and features of CP activation (complement‐fixing donor‐specific antibodies [DSA] and/or C4d deposition in kidney biopsies) were treated with four weekly doses of sutimlimab (60 mg/kg; Supporting Information S2). The present analysis was approved by the ethics committee of the Medical University of Vienna (EK No. 2126/2022).

### 2.2 Sampling and Measurements

Serum for the functional assessment of the classical complement pathway was collected at the indicated time points and measured as previously described [14, 15]; the procedure is also outlined in the Supporting Information S6: Materials and methods section. In brief, potent CP‐activating human leukocyte antigen (HLA) antibodies bound to HLA‐coated beads were incubated with test sera, and the deposited C3 complement split product C3d was measured using Luminex technology. Quantitative real‐time PCR of TTV load was performed on Days 0 and 14 (end‐of‐trial) in part A, Days 0, 14, and 35 (end‐of‐trial) in part B, and Days 0, 14, 36, and 50 (end‐of‐trial) in part C, as previously described methodologically [5] and summarized in the Supporting Information S6: Materials and methods.

### 2.3 Statistical Methods

For binary data, absolute numbers and frequencies in percentages were calculated. Continuous data were summarized by medians and the first and third quartiles. The primary analysis comprised a correlation analysis between classical complement activity (C3d log_10_ mean fluorescence intensity [MFI]) and TTV load (log_10_ c/mL) in the total study collective (184 data points: 48 study subjects from cohorts 1–7 with two time points, 16 subjects from cohorts 8 and 9 with three time points, and 10 transplant patients with four time points) and the healthy population only (144 data points: 48 study subjects from cohorts 1–7 with two time points and 16 subjects from cohorts 8 and 9 with three time points). For this, a linear regression and Pearson's correlation were calculated. For subgroup analysis, TTV loads were compared between different time points using the Wilcoxon signed‐rank test or the Friedman test, as appropriate. A two‐sided *p* < 0.05 was considered significant, and a *p *< 0.1 was regarded as a trend. All analyses were performed using SPSS software version 26 (IBM, Armonk, NY, USA). Figures 2, 3, 4 were drawn using the Python programming language version 3.10.13 relying on the packages matplotlib and seaborn.

## 3 Results

The study flow charts and baseline characteristics of participants in the clinical trial evaluating sutimlimab are detailed in Supporting Information S1, Supporting Information S2, and Supporting Information S6: Tables 1 and 2. In this trial, sutimlimab was previously demonstrated to effectively suppress complement activity in a dose‐dependent manner as measured by the deposition of C3d (a surrogate marker of the activity of the classical complement pathway) in both healthy volunteers and kidney transplant patients [14, 15] (Supporting Information S3, Supporting Information S4, and Supporting Information S5).

**Figure 1:**
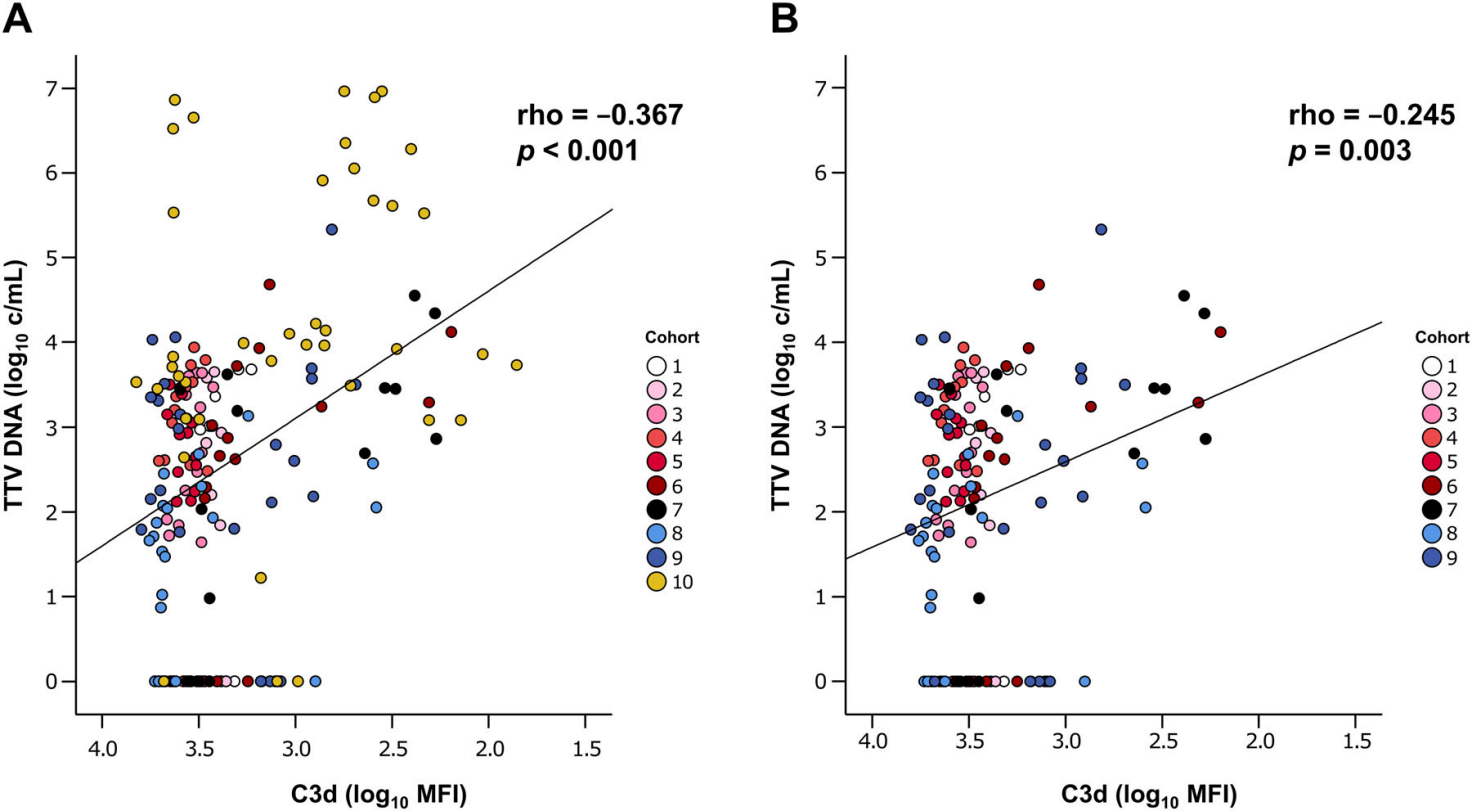
Correlation of complement activity and TTV load. The relation of the logarithmic values of C3d deposition as a surrogate of complement activity to the logarithmic copy number of TTV is shown in scatter plots. (A) In data set 1, all study participants and all time points were included in the analysis (184 data points). (B) In data set 2, the transplant recipients were excluded (144 data points). The contributions of the cohorts are visualized by color‐coding. Pearson's correlation coefficient (ρ) was calculated. C3d, complement component 3d; MFI, mean fluorescence intensity; TTV, Torque Teno virus.

In the present study, the impact of classical complement pathway inhibition on TTV load was analyzed. Linear regression and correlation analysis showed an association between classical complement activity and TTV load, with an increase in TTV load of 1.50 log_10_ c/mL per log decrease in C3d MFI in the total cohort (Pearson's *ρ* = −0.367, *p *< 0.001, *n* = 184; Figure 1A). To prevent the potential confounding of this result by immunosuppression, the analysis was repeated, including only healthy volunteers treated with sutimlimab and placebo and excluding data from kidney transplant recipients. This revealed a TTV load increase of 1.01 log_10_ c/mL per log decrease in C3d MFI (Pearson's *ρ* = −0.245, *p *= 0.003, *n* = 144; Figure 1B). In addition, sensitivity analyses excluding potentially uninfected individuals and considering alternative PCR limits of detection were performed (Supporting Information S6: Table 3).

**Figure 2:**
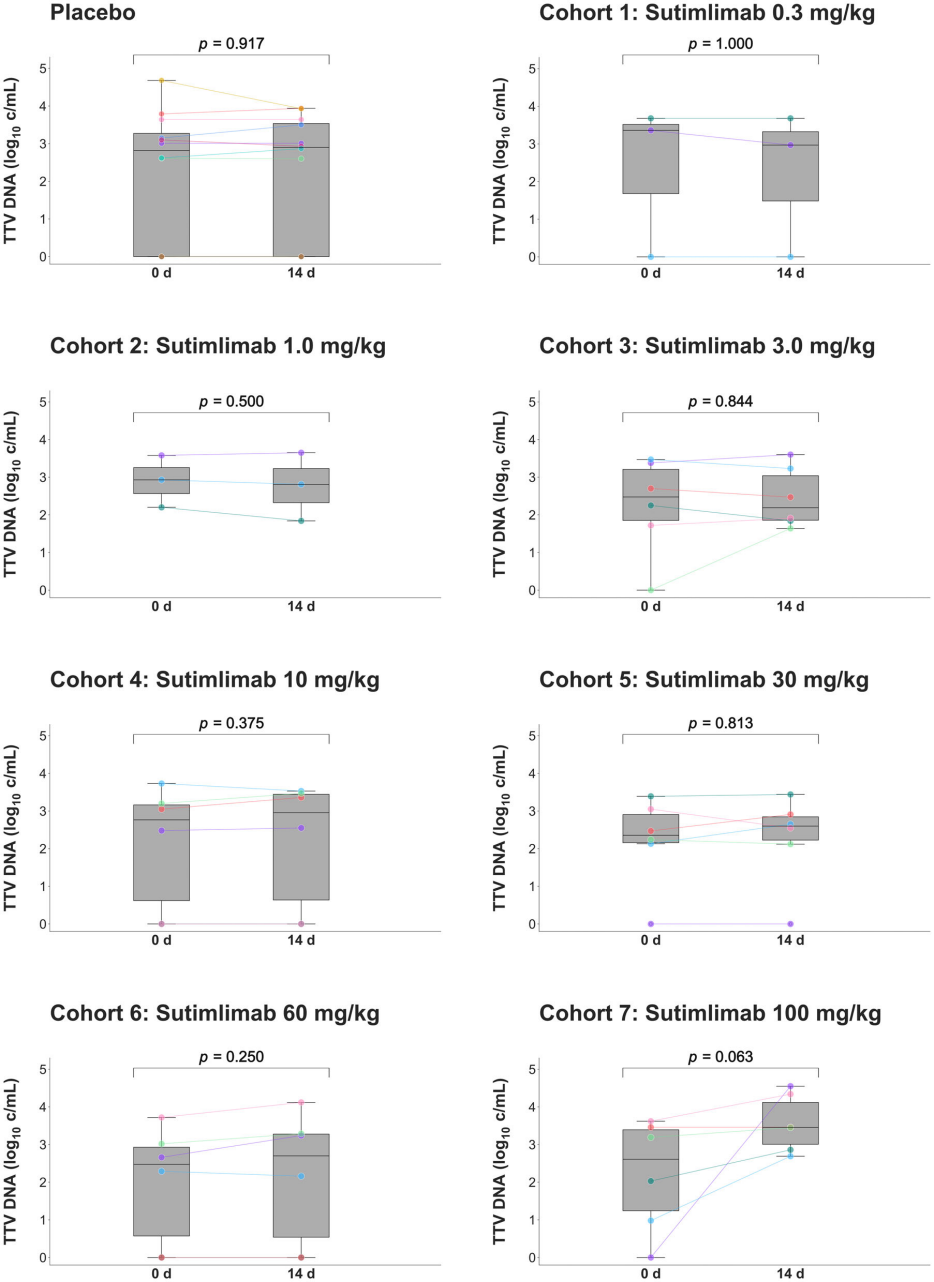
TTV load in cohorts receiving single doses of the anti‐C1s antibody sutimlimab. Logarithmic TTV loads at baseline and after 14 days are shown as line plots and boxplots demonstrating the median, interquartile range, and range. Outliers are indicated by small concentric circles. The results of each cohort (cohort 1 and 2: *n* = 3; cohort 3–7: *n* = 6) are presented in panels distinguished by the sutimlimab dosage; placebo subjects (*n* = 12) were removed from each cohort and are summarized in the top left panel. Individual subjects are distinguished by color‐coding. TTV load negative data points in cohorts 4 (*n* = 2), 6 (*n* = 2), and the placebo group (*n* = 4) are overlapping and appear as single subjects. The TTV loads were compared between the two time points using the Wilcoxon signed‐rank test for nonparametric paired data. c/mL, copies per milliliter; d, days; TTV, Torque Teno virus.

Next, cohorts restricted to single ascending doses of sutimlimab were analyzed (part A). Only at the highest dose of 100 mg/kg—the only dose that completely inhibited complement activity until Day 14 (Supporting Information S3)—a trend toward TTV load increase 14 days after administration was observed in healthy volunteers (Day 0: 2.9 log_10_ c/mL [interquartile range (IQR) 0.9–3.5], Day 14: 3.5 log_10_ c/mL [IQR 2.8–4.4]; *p *= 0.063, *n* = 6; Figure 2).

**Figure 3:**
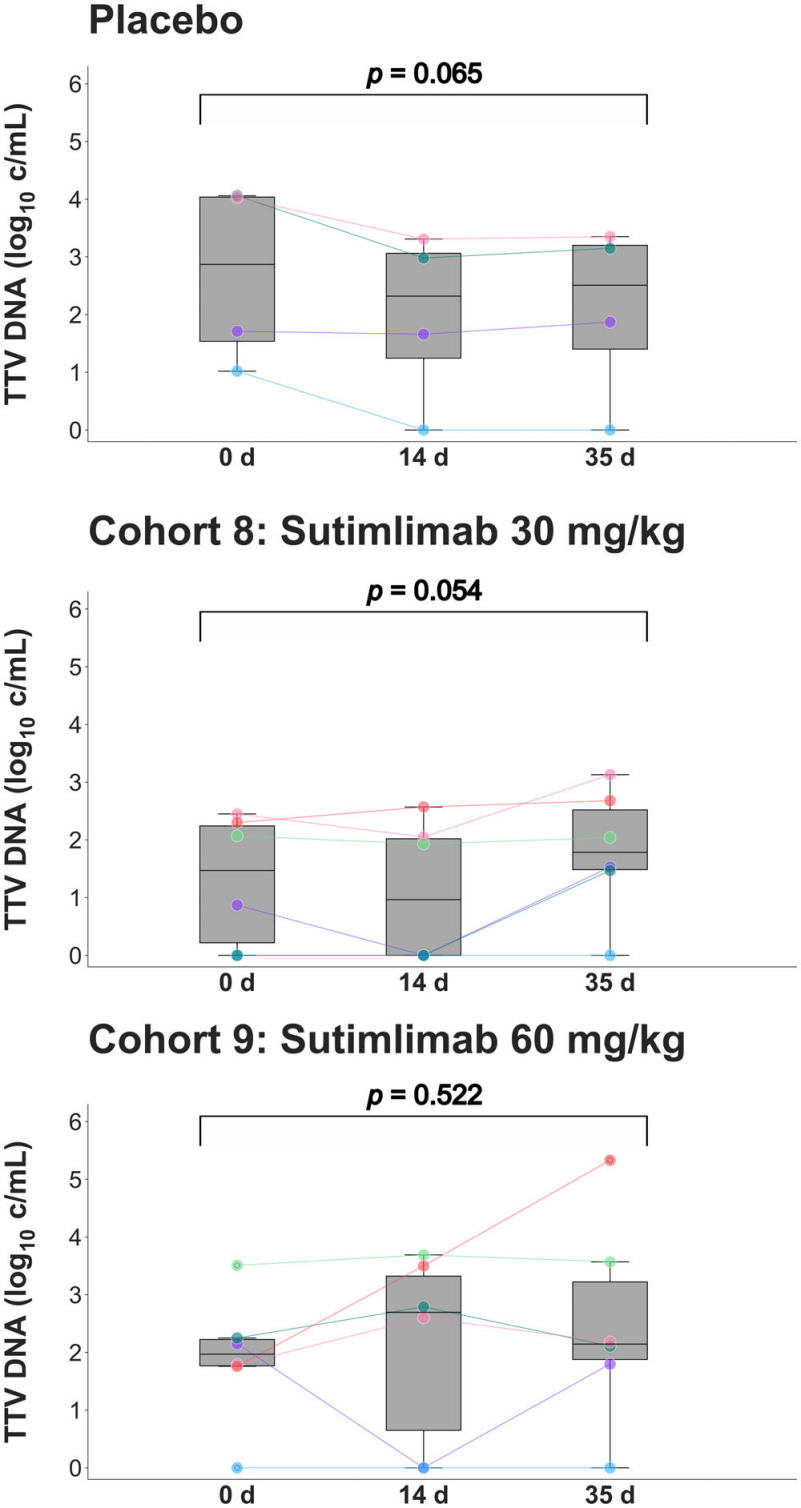
TTV load in cohorts receiving multiple doses of the anti‐C1s antibody sutimlimab. Subjects received four weekly doses of sutimlimab between Days 0 and 21. Logarithmic TTV loads at baseline and after 14 and 35 days are shown as line plots and boxplots demonstrating the median, interquartile range, and range. Outliers are indicated by small concentric circles. The results of each cohort (cohort size: *n* = 6) are presented in panels distinguished by sutimlimab dosage; placebo subjects (*n* = 4) were removed from each cohort and are summarized in the top panel. Individual subjects are distinguished by color coding. The TTV loads were compared between the three time points using the Friedman test, an extension of the Wilcoxon signed‐rank test for more than two comparisons. c/mL, copies per milliliter; d, days; TTV, Torque Teno virus.

In a further step, the cohorts of healthy volunteers who received multiple lower doses of either 30 or 60 mg/kg sutimlimab four times over 21 days (part B) were examined (Supporting Information S4). Although a statistical trend toward an increase in TTV load was calculated in the 30 mg/kg group (Day 0: 1.8 log_10_ c/mL [IQR 0–2.3], Day 35: 1.9 log_10_ c/mL [IQR 1.3–2.8]; *p *= 0.054, *n* = 6; Figure 3), the absolute differences in median TTV load were minimal in both groups (60 mg/kg group: Day 0: 2.0 log_10_ c/mL [IQR 1.6–3.0], Day 35: 2.1 log_10_ c/mL [IQR 1.7–4.8]; *p *= 0.522, *n* = 6; Figure 3).

**Figure 4:**
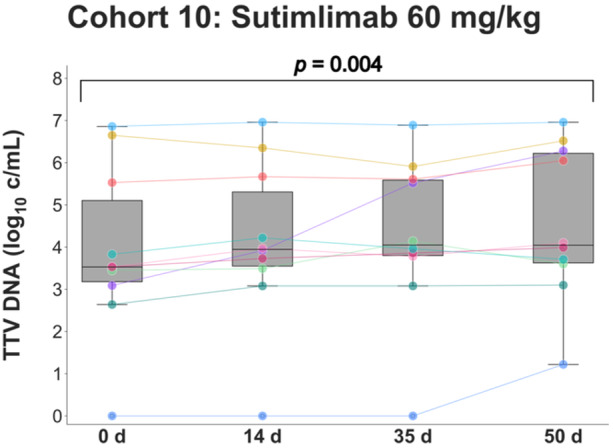
TTV load in kidney transplant patients receiving multiple doses of the anti‐C1s antibody sutimlimab. Ten patients with antibody‐mediated rejection received four weekly administrations of 60 mg/kg sutimlimab between Days 1 and 22 following an initial safety test dose of 10 mg/kg on Day 0. Logarithmic TTV loads at baseline and after 14, 36, and 50 days are shown as line plots and boxplots demonstrating the median, interquartile range, and range. Outliers are indicated by small concentric circles. Individual subjects are distinguished by color coding. TTV load was compared between the four time points using the Friedman test (*p* = 0.004). c/mL, copies per milliliter; d, days; TTV, Torque Teno virus.

Finally, TTV load in the 10 immunosuppressed kidney transplant recipients who received four weekly doses of 60 mg/kg sutimlimab over 21 days (part C) was assessed (Supporting Information S5). Here, an increase in TTV load between baseline and Day 50 was detected (Day 0: 3.5 log_10_ c/mL [IQR 3.0–6.1], Day 50: 4.1 log_10_ c/mL [IQR 3.5–6.4]; *p *= 0.004; Figure 4).

## 4 Discussion

This is the first study to investigate the role of the host's complement system in the context of TTV load control. Within a first‐in‐human trial testing sutimlimab—an antibody neutralizing classical complement pathway activity—the effect of complement inhibition on TTV load could be analyzed. The primary finding of the present study was the identification of an increase in TTV load following complement inhibition, suggesting a role for the complement system in TTV replication control.

This effect was demonstrated in the overall cohort including healthy volunteers and immunosuppressed individuals. This finding was also confirmed in a cohort restricted to a healthy population, suggesting an effect of complement inhibition on TTV load regardless of conventional post‐transplant immunosuppression.

When the effect was further dissected by analyzing individual cohorts of healthy volunteers, statistical trend toward an association between TTV load increase and complement inhibition was detected when sutimlimab was administered at the highest single dose and multiple lower doses. Only the immunosuppressed kidney transplant cohort receiving repeat doses exhibited a statistically significant TTV load increase after almost 2 months of observation. In this cohort, the TTV load was still increasing at Day 50, even though complement activity was already rebounding at that point. This finding aligns with previous observations showing TTV load to fully reflect changes in immunosuppression only after 2 months [16, 17]. It is also conceivable that exposure to sutimlimab for even longer than 21 days might lead to a larger increase in TTV load.

In some individuals, particularly in study part B, the baseline TTV load was low and increased only marginally despite complement activity being adequately suppressed by the administration of high and multiple doses of sutimlimab. In the 30 mg/kg group (cohort 8) a statistical trend toward a TTV load increase was calculated. In light of the small absolute changes in TTV load and increasing inaccuracy regarding quantification of low TTV loads, such a result might reflect the use of a nonparametric paired test, which rather counts and compares the increases versus the decreases of the paired samples than weighing clinically meaningful absolute differences. It can be hypothesized that most of these individuals in part B were never infected with TTV or, at least, lack complement‐activating anti‐TTV antibodies, and, therefore, neutralization of the classical complement pathway does not lead to a significant difference in TTV loads. To identify and stratify these study participants, a serological test covering immunoglobulin reactivities against all TTV strains would be required, which is not yet available [11].

One concern might be the seemingly small contribution of blocking the classical complement pathway, compared to the several log steps increase in TTV load observed after the introduction of immunosuppression targeting cellular immunity, for example in the context of solid organ transplantation. In this respect it is important to mention that the classical complement pathway represents only one part of the humoral immune system; the presence of neutralizing anti‐TTV antibodies themselves or the alternative and lectin pathways of the complement system, could also contribute significantly to the immune control of TTV.

The main limitations of the present study are, on the one hand, that the clinical trial was not powered to detect differences in TTV load, which is primarily reflected in the small number of cases available to analyze each cohort. On the other hand, the observation time in healthy volunteers was short, which did not allow for detecting a further increase in TTV load or the time point of return to baseline. A major strength of this study is the unique opportunity to analyze TTV load in participants of a placebo‐controlled, first‐in‐human trial consisting of both healthy subjects and immunosuppressed individuals.

The study data provide evidence for an association between TTV load and classical complement pathway activity. Complement inhibition is a potent therapeutic option for hematological [18], nephrological [19], and neurological [20] diseases, with many emerging substances on the horizon [21]. Therefore, the potential of TTV assessment for immunologic monitoring in these applications could be of interest in the near future.

Another aspect of CP activation, namely, the presence of anti‐TTV antibodies, as well as other activation pathways of the complement system (alternative and lectin pathways) may also play a role in TTV load control and need to be tested in future studies.

## Author Contributions

**Sebastian Kapps:** investigation, data curation, visualization, writing–original draft. **Jakob Mühlbacher:** investigation, methodology, writing–review and editing. **Dorian Kulifaj:** investigation, methodology, writing–review and editing. **Sophie Courjal:** investigation, methodology, writing–review and editing. **Farsad Eskandary:** data curation, writing–review and editing. **Martin Schiemann:** conceptualization, writing–review and editing. **Bernd Jilma:** resources, writing–review and editing. **Georg A. Böhmig:** resources, writing–review and editing. **Gregor Bond:** conceptualization, funding acquisition, investigation, project administration, resources, supervision, validation, writing–original draft. **Markus Wahrmann:** conceptualization, investigation, data curation, formal analysis, methodology, project administration, validation, visualization, writing–original draft.

## Conflict of Interest

Gregor Bond received fees for a talk at a scientific conference and the preparation of communication material on the TTV R‐GENE PCR from bioMérieux.

Dorian Kulifaj and Sophie Courjal are employees of bioMérieux.

## Supporting information

Supporting information.

Supporting information.

Supporting information.

Supporting information.

Supporting information.

Supporting information.

## Data Availability

The data that support the findings of this study are available from the corresponding author upon reasonable request.
