# Supplementary figures and images for: Torque Teno Virus Control by the Classical Pathway of Complement Activation—A Retrospective Analysis From a First‐in‐Human Trial Utilizing Sutimlimab
Source: J Med Virol. 2024 Nov 6;96(11):e70039. doi: 10.1002/jmv.70039 (PMC11600468; doi:10.1002/jmv.70039)

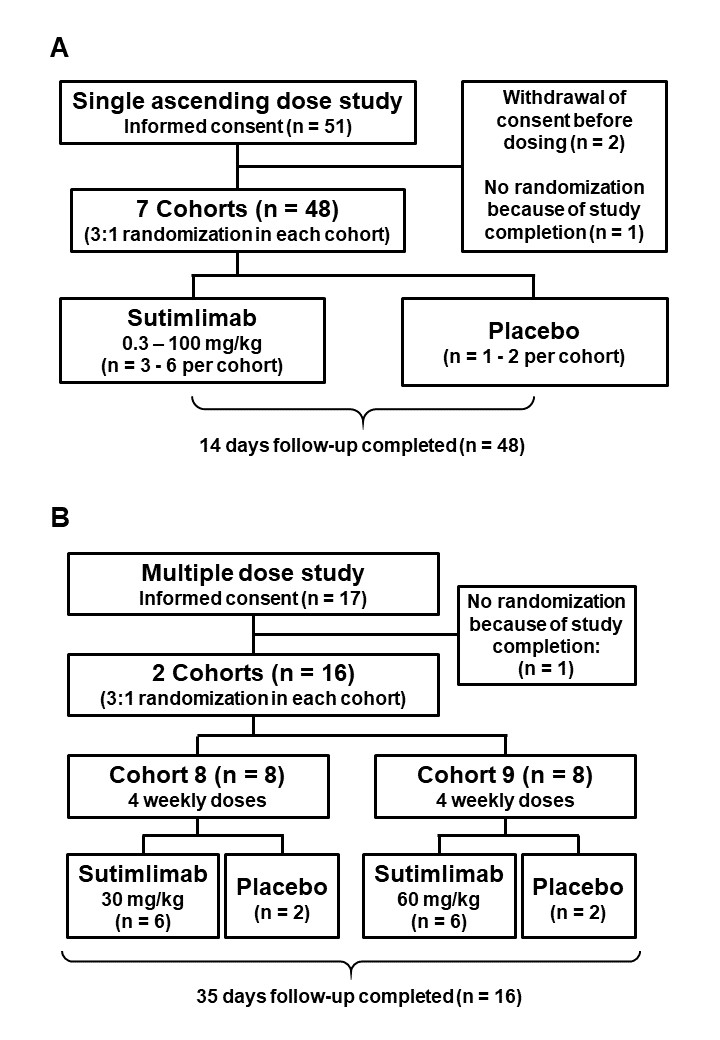

Supplement: Supplementary file 1 — Supporting information. [file JMV-96-e70039-s002.png]

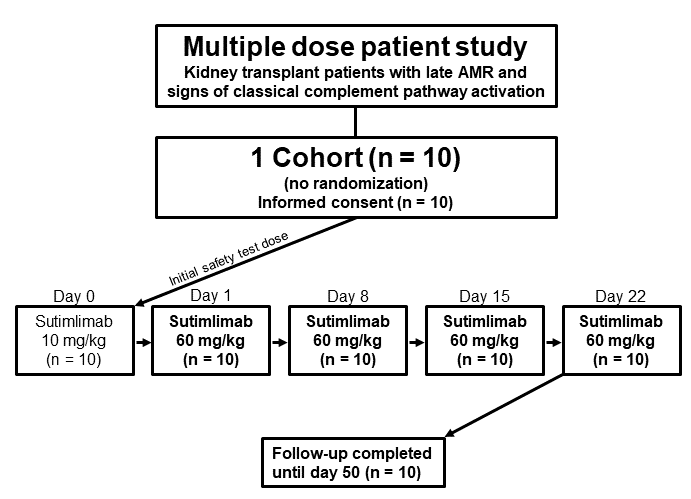

Supplement: Supplementary file 2 — Supporting information. [file JMV-96-e70039-s003.png]

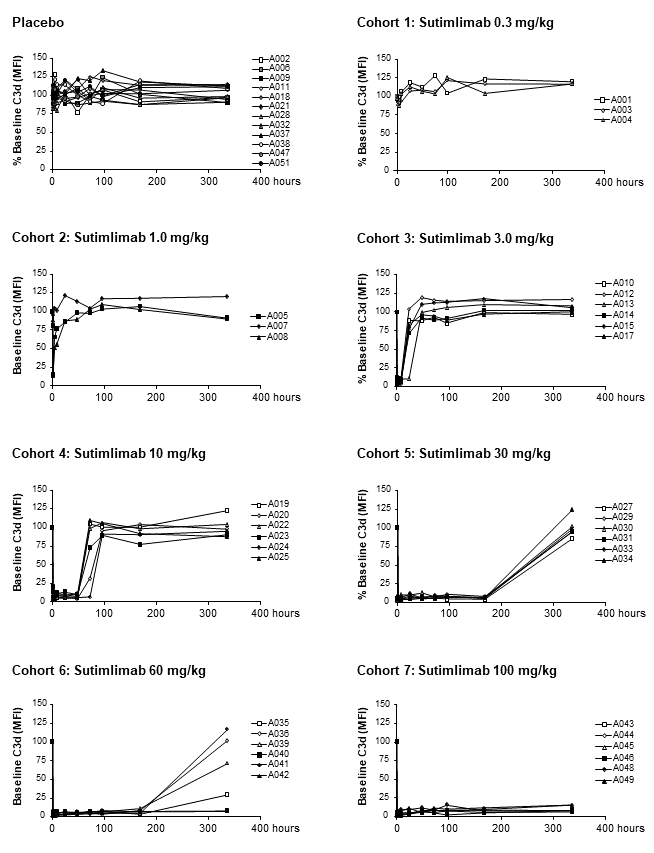

Supplement: Supplementary file 3 — Supporting information. [file JMV-96-e70039-s001.png]

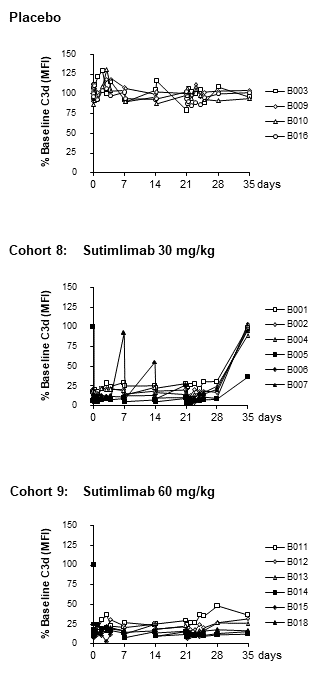

Supplement: Supplementary file 4 — Supporting information. [file JMV-96-e70039-s005.png]

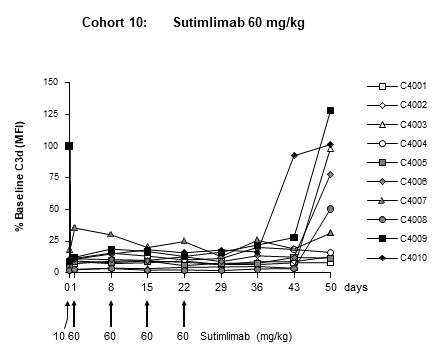

Supplement: Supplementary file 5 — Supporting information. [file JMV-96-e70039-s004.png]
