# Supplementary material for: Torque Teno Virus Control by the Classical Pathway of Complement Activation—A Retrospective Analysis From a First‐in‐Human Trial Utilizing Sutimlimab
Source: J Med Virol. 2024 Nov 6;96(11):e70039. doi: 10.1002/jmv.70039 (PMC11600468; doi:10.1002/jmv.70039)
Supplement: Supplementary file 6 — Supporting information. [file JMV-96-e70039-s006.docx]

**SUPPLEMENTAL MATERIAL**

to

**Torque Teno virus control by the classical pathway of complement activation – a retrospective analysis from a first-in-human trial utilizing sutimlimab**

Sebastian Kapps, Jakob Mühlbacher, Dorian Kulifaj, Sophie Courjal, Farsad Eskandary, Martin Schiemann, Bernd Jilma, Georg A. Böhmig, Gregor Bond, Markus Wahrmann

**Correspondence:** Gregor Bond, MD, PhD; Phone: +43-1-40400-72530; E-mail: gregor.bond@meduniwien.ac.atgregor.bond@meduniwien.ac.at

**Running head:** TTV during classical complement inhibition

**Supplemental materials and methods**

*Inclusion and exclusion criteria*

All healthy volunteers had to be ≥ 18 years of age, without chronic illness, and without infection within the past 30 days. Female participants had to be non-pregnant/non-lactating and postmenopausal or surgically sterilized or willing to use highly effective methods of birth control throughout the study and for 30 days after the end-of-study visit. At least 14 days before the first dose of sutimlimab, all participants had to complete a vaccination program against encapsulated bacteria (*Neisseria meningitidis*, *Haemophilus influenzae*, and *Streptococcus pneumoniae*). Kidney transplant patients had to have a functioning allograft with an estimated glomerular filtration rate (eGFR) ≥ 20 mL/min/1.73m^2^ ≥ 180 days post-transplantation, with a biopsy-proven antibody mediated rejection (AMR) showing signs of classical pathway (CP) activation (complement-fixing donor-specific antibody [DSA] and/or C4d deposition). Furthermore, they received ciprofloxacin prophylaxis (250 mg orally twice a day) throughout the study.

*Classical complement activity measurement by complement component 3 split product (C3d) deposition analysis*

The impact of sutimlimab-induced C1s inhibition on complement activation was investigated in healthy volunteers and kidney transplant patients through ex vivo experiments using potent CP-triggering human leukocyte antigen (HLA) antibodies bound to HLA-coated beads. First, LABScreen Mixed beads (One Lambda, Canoga Park, CA, USA) were incubated with pooled heat-inactivated sera from three highly sensitized end-stage kidney disease patients who had been immunized through prior kidney transplants. These sera exhibited > 99% immunoglobulin G (IgG) virtual panel reactivity in single antigen bead assays. Following incubation and washing, the antibody-coated beads were exposed to either test sera or a heat-inactivated (negative control) serum. After washing, the beads were stained with biotinylated anti-C3d antibody (30 minutes, final concentration: 4 μg/mL; Quidel, San Diego, CA, USA) and phycoerythrin-conjugated streptavidin (30 minutes, final concentration: 1 μg/mL; eBioscience, San Diego, CA, USA) before analysis on a Luminex 200 flow analyzer (Luminex Corporation, Austin, TX, USA). The results were presented as normalized C3d mean fluorescent intensity (MFI), calculated by subtracting the raw MFI (obtained with the heat-inactivated serum) from the test serum MFI. The mean value of the normalized C3d MFI was calculated for each test serum across 12 HLA class I and four of the five HLA class II bead populations within the panel (excluding one HLA class II bead population [ID25] due to consistently negative C3d staining with MFI < 100).

In the single-ascending-dose part of the trial (part A), serum was collected at 11 time points (1 hour before and 0.5, 1, 4, 8, and 24 hours after infusion and on post-infusion days 2, 3, 4, 7, and 14 [end-of-trial visit]). During the multiple-ascending-dose part (part B), 24 consecutive samples were drawn per subject (day 0: 1 hour before and 0.5, 1, 4, and 8 hours after the first infusion; days 1, 2, 3, and 4; day 7: 1 hour before and 4 hours after the second infusion; day 14: 1 hour before and 4 hours after the third infusion; day 21: 1 hour before and 0.5, 1, 4, and 8 hours after the fourth infusion; and days 22, 23, 24, 25, 28, and 35 [end-of-trial visit]). In part C, serum samples from transplant patients were collected 12 times: on day 0 (1 hour before administering the initial 10 mg/kg test dose of sutimlimab); 1 hour before each full dose of sutimlimab on days 1, 8, 15, and 22; and on days 29, 36, 43, and 50 (end-of-study visit). All sera were aliquoted and stored at −80 °C until complement split product deposition analysis, strictly avoiding repeated freezing and thawing.

*Quantitative real-time polymerase chain reaction (qPCR)*

Prior to nucleic acid extraction, Internal Control 2 (for checking the inhibition of the PCR) and the negative control of the TTV R-GENE^®^ kit (in vitro diagnostics regulation [IVDR]-marked, bioMérieux, Marcy-l'Étoile, France) were added to all serum samples. Then, nucleic acid was extracted from 200 µL of the samples (serum and negative control) and eluted in 50 µL elution buffer using the EMAG^®^ platform (bioMérieux, Marcy-l'Étoile, France) as recommended by the manufacturer. TTV quantitation using the TTV R-GENE^®^ kit was performed according to the manufacturer’s instructions (bioMérieux). Briefly, 10 µL of extracted DNA was added to 15 µL of the ready-to-use amplification mixture. In each run, one sensitivity control and four quantification standards were included. Thermal cycling was performed in a CFX96 cycler (Biorad, Hercules, CA, USA) and was started with a denaturation step at 95 °C for 15 minutes, followed by 45 cycles at 95 °C for 10 seconds for denaturation and 60 °C for 40 seconds for combined annealing and elongation. The results were determined using the range of quantification standards, reported as c/mL, and finally log-transformed. The IVDR (in vitro diagnostics regulation) certified limit of detection and the lower limit of quantification in serum of this assay is 2.4 log_10_ c/mL. However, due to this analysis being research use only (RUO) and the overall low number of subjects in the study, the assay’s capabilities were extended in accordance with the manufacturer. As presented at the 43rd Réunion Interdisciplinaire de Chimiothérapie Anti-Infectieuse (RICAI) (Bonabaud M. et al., poster P-176 Dec 2023), in a serial dilution of TTV 8 in serum down to a theoretical 79 c/ml, a hit rate of 3/3 replicates was achieved. Similarly, internal tests performed by the manufacturer showed a hit rate of 20/20 replicates in a dilution of a linearized TTV 6 plasmid in serum down to a theoretical 63 c/mL. Maggi et al. showed that the TTV R-GENE^®^ PCR produced reliable results in plasma even at 12 c/mL (*Journal of Medical Virology*, doi: https://doi.org/10.1002/jmv.25488). Therefore, according to the study’s RUO approach, only result outputs given as 0 c/mL were regarded as negative. The qPCR of TTV DNA was analyzed at selected time points: on days 0 and 14 (end-of-trial) in part A, on days 0, 14, and 35 (end-of-trial) in part B, and on days 0, 14, 36, and 50 (end-of-trial) in part C

**Supplemental results**

**Supplemental Table 1. Baseline characteristics of healthy volunteers.**

| **Parameters** | **Total (n = 64)** | **Sutimlimab (n = 48)** | **Placebo (n = 16)** |
| --- | --- | --- | --- |
| **Baseline data recorded on day 0** |  |  |  |
| Female sex, n (%) | 26 (40.6) | 22 (45.8) | 4 (25) |
| Age (y), median (IQR) | 30.5 (26.6–36.3) | 30.4 (26.3–36.3) | 30.5 (27.6–38.1) |
| Body mass index, median (IQR) | 23.1 (20.9–24.9) | 22.6 (21.1–24.7) | 23.9 (20.6–25.4) |
| **CP activity/level^a^ at day 0** |  |  |  |
| CH50 activity (%), median (IQR) | 114 (102–126) | 115 (103–127) | 109 (100–123) |
| C4 (mg/dL), median (IQR) | 17 (15–22) | 17 (15–21) | 18 (16–23) |
| C3d (log_10_ MFI), median (IQR) | 3.5 (3.4–3.7) | 3.5 (3.4–3.6) | 3.5 (3.5–3.7) |

y, years; IQR, interquartile range; CP, classical pathway; CH50, hemolytic complement activity; C4, complement factor 4; C3d, complement factor C3d; MFI, mean fluorescence intensity. Baseline differences between groups were non-significant (*p* > 0.05).

^a^Normal ranges: 10–40 mg/dL (C4), 0–140% (CH50 activity).

**Supplemental Table 2. Baseline characteristics of the transplant cohort.**

| **Parameters** |  | **Total (n = 10)** |
| --- | --- | --- |
| **Variables recorded at the time of transplantation** |  |  |
| Female sex, n (%) |  | 4 (40) |
| Age (y), median (IQR) |  | 45.4 (38.1–63.5) |
| Body mass index, median (IQR) |  | 28.1 (25.6–30.5) |
| Live donor, n (%) |  | 2 (20) |
| ABO-incompatible live donor transplant, n (%) |  | 1 (10) |
| Prior kidney transplant, n (%) |  | 3 (30) |
| *Immunosuppression at the time of transplantation* |  |  |
| Induction with IL-2R antibody, n (%) |  | 4 (40) |
| Induction with anti-thymocyte globulin, n (%) |  | 3 (30) |
| Peri-transplant immunoadsorption |  | 3 (30) |
| Tacrolimus-based triple immunosuppression, n (%) |  | 9 (90) |
| Cyclosporine A-based triple immunosuppression, n (%) |  | 1 (10) |
| **Variables recorded at the time of study inclusion** |  | |
| Time to study inclusion (y), median (IQR) |  | 4.3 (2.9–8.0) |
| Age (y), median (IQR) |  | 51.5 (46.4–66.3) |
| eGFR (ml/min/1.73 m^2^), median (IQR) |  | 46 (27–61) |
| Urinary protein/creatinine ratio (mg/g), median (IQR) |  | 399 (162–861) |
| Serum creatinine (mg/dl), median (IQR) |  | 1.7 (1.4–2.4) |
| CP activity/level^a^ at the time of study inclusion |  |  |
| CH50 activity (%), median (IQR) |  | 140 (140–140) |
| C4 (mg/dL), median (IQR) |  | 23 (19–27) |
| C3d (log_10_ MFI), median (IQR) |  | 3.6 (3.6–3.7) |
| *Immunosuppression at the time of study inclusion* |  |  |
| Tacrolimus, MPA, glucocorticoid, n (%) |  | 9 (90) |
| Cyclosporine A, MPA, glucocorticoid, n (%) |  | 1 (10) |

y, years; IQR, interquartile range; CP, classical pathway; IL-2R, interleukin-2 receptor; CH50, hemolytic complement activity; C4, complement factor 4; C3d, complement factor C3d; MFI, mean fluorescence intensity; eGFR, estimated glomerular filtration rate; MPA, mycophenolic acid.

^a^Normal ranges: 10–40 mg/dL (C4), 0–140% (CH50 activity); values above 140% percent were set to 140.

*Sensitivity Analyses*

For the primary analysis all TTV values > 0 c/mL were regarded as positive. In addition, subjects who were TTV negative at all the time points were included. To analyze the impact of this selection strategy on the robustness, sensitivity analyses were performed excluding the 12 study subjects (26 data points) who were negative at all the time points, and applying the IVDR limit of detection (2.4 log10 c/mL, 34 data points set to zero), and the lowest copy number with a known hit rate of 100% (63 c/mL, 12 data points set to zero). Additionally, two analyses were performed excluding all the data points below the respective alternative limits of detection (2.4 log10 and 63 c/mL; Supplemental Table 3). The slope of the TTV load increase of our primary analysis (k=1.50) was within the range of the sensitivity analyses (0.80-1.87), providing evidence for its robustness.

**Supplemental Table 3. Sensitivity analyses.**

| **Analysis** | **K** | **LoD  (c/mL)** | **Data points included (n)** | **Pearson’s ρ** | ***p*** |
| --- | --- | --- | --- | --- | --- |
| **Characteristics of analysis** |  |  |  |  |  |
| Benchmark analysis (Figure 1A) | 1.50 | > 0 | 184 | −0.367 | < 0.001 |
| Individuals negative at all the time points excluded | 1.22 | > 0 | 158 | −0.369 | < 0.001 |
| LoD set to 250 c/mL, data points < LoD set to 0 c/mL | 1.87 | 250 | 184 | −0.394 | < 0.001 |
| LoD set to 63 c/mL, data points < LoD set to 0 c/mL | 1.67 | 63 | 184 | −0.386 | < 0.001 |
| LoD set to 250 c/mL, data points < LoD excluded | 0.80 | 250 | 113 | −0.346 | < 0.001 |
| LoD set to 63 c/mL, data points < LoD excluded | 0.98 | 63 | 135 | −0.385 | < 0.001 |

k, log TTV load increase per log C3d decrease; LoD, limit of detection; c/mL, copies per milliliter;

**Supplemental figure legends**

**Supplemental Figure 1. (A) Study flow chart of the anti-C1s antibody sutimlimab in a single-ascending-dose study.** Forty-eight healthy volunteers were randomized 3:1 into seven cohorts (size: n = 4 or 8) to receive a single administration of ascending doses of sutimlimab (0.3, 1, 3, 10, 30, 60, or 100 mg/kg body weight) or placebo. (**B)** **Study flow chart of the anti-C1s antibody sutimlimab in a multiple-dose study with two different dosages.** Sixteen healthy volunteers were randomized 3:1 into two cohorts (size: n = 8) to receive four weekly administrations of 30 mg/kg (cohort 8) or 60 mg/kg sutimlimab (cohort 9) or placebo.

**Supplemental Figure 2. Study flow chart of the anti-C1s antibody sutimlimab in transplant patients.** Ten patients with antibody-mediated rejection received four weekly administrations of 60 mg/kg sutimlimab (cohort 10) after an initial safety test dose of 10 mg/kg.

**Supplemental Figure 3. Complement activity in cohorts of healthy volunteers who received single doses of the anti-C1s antibody sutimlimab.** The course of complement activity is presented as a percentage of baseline C3d deposition. Panels are depicted depending on the administered dose of sutimlimab. Subjects from all cohorts who received the placebo are grouped in the top left panel. Note: all panels start with a complement activity of 100% (indicated by a singular visible data point at time point zero), which drops immediately in treated subjects (sutimlimab ≥ 1.0 mg/kg) within a few minutes and rebounds delayed, depending on the concentration of sutimlimab.

C3d, complement component 3d; MFI, mean fluorescence intensity

**Supplemental Figure 4. Complement activity in cohorts of healthy volunteers who received multiple doses of the anti-C1s antibody sutimlimab.** Subjects received four weekly doses of sutimlimab between days 0 and 21. The course of complement activity is presented as a percentage of baseline C3d deposition. Panels are depicted depending on the administered dose of sutimlimab. Subjects from all cohorts who received the placebo are grouped in the top panel. Note: all panels start with a complement activity of 100% at time point zero, which drops immediately in treated subjects within a few minutes.

C3d, complement component 3d; MFI, mean fluorescence intensity

**Supplemental Figure 5. Complement activity in a cohort of kidney transplant patients treated with multiple doses of the anti-C1s antibody sutimlimab.** Ten patients with antibody-mediated rejection received four weekly administrations of 60 mg/kg sutimlimab between days 1 and 22 after an initial safety test dose of 10 mg/kg on day 0. The course of complement activity is presented as a percentage of baseline C3d deposition. Note: the course of complement activity of all patients starts at 100% (indicated by a singular visible data point at time point zero), which drops immediately within a few minutes.

C3d, complement component 3d; MFI, mean fluorescence intensity
